# Objects Mental Rotation under 7 Days Simulated Weightlessness Condition: An ERP Study

**DOI:** 10.3389/fnhum.2017.00553

**Published:** 2017-12-06

**Authors:** Hui Wang, Jiaobo Duan, Yang Liao, Chuang Wang, Hongzheng Li, Xufeng Liu

**Affiliations:** ^1^Department of Medical Psychology, Fourth Military Medical University, Xi'an, China; ^2^Aviation Psychology Center, Institute of Aviation Medicine, Air Force, Beijing, China; ^3^Mental Health Center, 303 Hospital of PLA, Nanning, China

**Keywords:** head down tilt (HDT) bed rest, simulated microgravity, mental rotation, event related potentials

## Abstract

During the spaceflight under weightlessness condition, human's brain function may be affected by the changes of physiological effects along with the distribution of blood and body fluids to the head. This variation of brain function will influence the performance of astronauts and therefore create possible harm to flight safety. This study employs 20 male subjects in a 7-day−6° head-down tilted (HDT) bed rest model to simulate physiological effects under weightlessness condition, and use behavioral, electrophysiological techniques to compare the changes of mental rotation ability (MR ability) before and after short-term simulated weightlessness state. Behavioral results suggested that significant linear relationship existed between the rotation angle of stimuli and the reaction time, which means mental rotation process do happen during the MR task in simulated weightlessness state. In the first 3 days, the P300 component induced by object mental rotation followed the “down-up-down” pattern. In the following 4 days it changed randomly. On HDT D2, the mean of the amplitude of the P300 was the lowest, while increased gently on HDT D3. There was no obvious changing pattern of the amplitude of P300 observed after 3 days of HDT. Simulated weightlessness doesn't change the basic process of mental rotation. The effect of simulated weightlessness is neural mechanism of self-adaptation. MR ability didn't bounce back to the original level after HDT test.

## Introduction

It is commonly believed that the individual cognitive and behavioral activities would be impaired in space environment due to the negative influential factors like microgravity, radiation, noise, fatigue, etc. (Eddy et al., 1998; Morphew, 2001; Palinkas, 2007; Sandal, 2007). Among those factors, microgravity can be considered as one of the most obvious and significant environmental difference between space environment and the earth environment. In this sense, microgravity-related studies were widely conducted in recent years and there were many significant findings (Grigoriev and Egorov, 1992). Researchers have proposed that microgravity can harm human's cardiovascular system, endocrine system, the central nerve system and other systems (Hirayanagi et al., 2004; Zayzafoon et al., 2005). In addition, the changes of central nerve system will further influence the vestibular function, the visual and sensory - motor system, which as a result would affect human's cognitive function (Newberg and Alavi, 1998). On the other hand, the cerebrovascular circulation system changes, such as, the increased blood pressure caused by the redistribution of body fluid, will certainly harm cognitive function in short or long term. Most relevant studies concentrate on the topic of spatial orientation (Benke et al., 1993; Kornilova, 1997; Leone, 1998; Manzey and Lorenz, 1998), object recognition (Koga, 2000), motion perception (McIntyre et al., 1998; Pozzo et al., 1998; Kelly et al., 2005), as well as some high-level cognitive functions like learning and memory, reasoning and calculation (Johnston and Dietlein, 1977; Manzey and Lorenz, 1998; Shehab and Schlegel, 2000).

During the flight under weightlessness condition, brain function will be affected by the physiological effects of the redistribution of blood and body fluids to the head, which may influence the performance of astronauts. This requires astronaut to have extraordinary spatial cognitive ability. Mental rotation (MR) was first revealed in behavioral experiments, which refers to a type of spatial ability in which a person imagines how an object or array would appear if rotated away from the presented orientation (Shepard and Metzler, 1971; Cooper and Shepard, 1973). The results of the experiments showed a linear relationship between the degree of angular disparity of the objects and the response time (RT) for rotations up to 180°. This finding suggests that mental manipulation of objects follows laws akin to those for real manipulation of physical objects. MR have been extensively studied in cognitive psychology during these four decades, with stimuli including images of two- or three-dimensional graphics (Jolicoeur, 1985, 1988, 1990; Schendan and Lucia, 2009; Wraga et al., 2010), letters like characters (Tarr and Pinker, 1989; Hu et al., 2013), body parts (Cooper and Shepard, 1975; Horst et al., 2012), scenes (Zacks et al., 2003; Dalecki et al., 2012), and chemical formula (Huang and Liu, 2012). Stimuli of objects MR are usually images of two- or three-dimensional graphics, and characters. Most people believe that in objects MR tasks, observers seem to use a rotate-then-match scheme, in details, which, they compare the stimuli with a visual imagery in their long-term memory entry (e.g., NM/LR task) or the imagery of the original stimuli (e.g., S1–S2 task), before making the judgment (Shepard and Metzler, 1971).

Event-related potentials (ERPs) analysis is an important tool to assess the time course of MR. Abundant research results have shown that the amplitude of ERPs recorded over the parietal cortex varies in accordance with the rotation angle; during the interval ranging from ~350 to 800 ms, the ERPs amplitude is more negative for characters rotated away from the upright position (Peronnet and Farah, 1989). It has been suggested that this slow negativity (Rotation-related negativity, RRN), which is overlapped on a broad positive deflection (the late positive complex, LPC), is specifically correlated with the mental rotation process (Peronnet and Farah, 1989; Wijers et al., 1989). Control experiments using various stimuli and task demands supported this interpretation (Heil, 2002). The amplitude of RRN increased with the increasing rotation angle of stimulus. Less negative amplitudes were recorded in subjects who perform better (i.e., faster) in the task (Riečanský and Jagla, 2008). RRN is independent of stimulus type, but the difference between stimuli is unreported (Riečanský et al., 2013). Two different amplitudes of RRN have been reported in the previous studies, and some researchers claimed that different RRN may be caused by different tasks used.

The results from previous researches and our latest studies indicated that the cognitive function changes of visual dorsal pathway caused by weightless physiological effect has its own self-adaptive mechanism, and the aging and balanced characteristics reflect the internal neural mechanism of self-adaptive ability. It has been proved that head-down tilt (HDT) bed rest can effectively simulate the physiological effects of body fluid redistribution caused by microgravity, which serves as a good model for further in-depth study. In this study, we intend to use HDT bed rest model to simulate physiological effects of weightlessness, using behavioral, electrophysiological techniques to compare the objects MR ability changes before and after 7-day simulated weightlessness state. The results provide new evidence in demonstrating the effect of weightless on cognitive function of visual dorsal pathway and its mechanism. The conclusion may have some value in the future theoretical researches and practical tasks of China's medium-and long-term manned space program and astronaut selection and training.

## Materials and methods

### Participants

Twenty male participants were recruited. The valid participants' mean age was 24 years, with a range from 20 to 32 years. All the participants were right-handed as measured by the Handedness Questionnaire (Annett, 1970). The participants reported no history of neurological injury, genetic mental disorders, or substance abuse. This study was approved by the Ethical Committee of the Fourth Military Medical University and all participants signed informed consent for experimental participation.

### HDT bed rest procedure

There were three periods during the experiment: the period prior to bed rest (HDT D0), the bed rest period (HDT D1-HDT D7) and the post bed rest period (7 days after bed rest, HDT D14). During the HDT bed rest, adequate water and food were supplied, but the subjects' heads were restricted to keep on the bed for the redistribution of the body fluids toward the head. The experimental room was air-conditioned, and the temperature was maintained around 22°C. All of the experimenters were well-educated with medical knowledge and skills, thus they could provide appropriate care for the subjects. All the subjects were required to remain in bed on their back and not allowed to seat upright or lift heads. The only gestures they could make were some simple ones via hands. Additionally, the subjects were paired, and they were allowed to communicate with each other and engage in recreational activities (such as, reading, watching television or movies, and using the internet) in their leisure time.

### Mental rotation task

**Figure 1:**
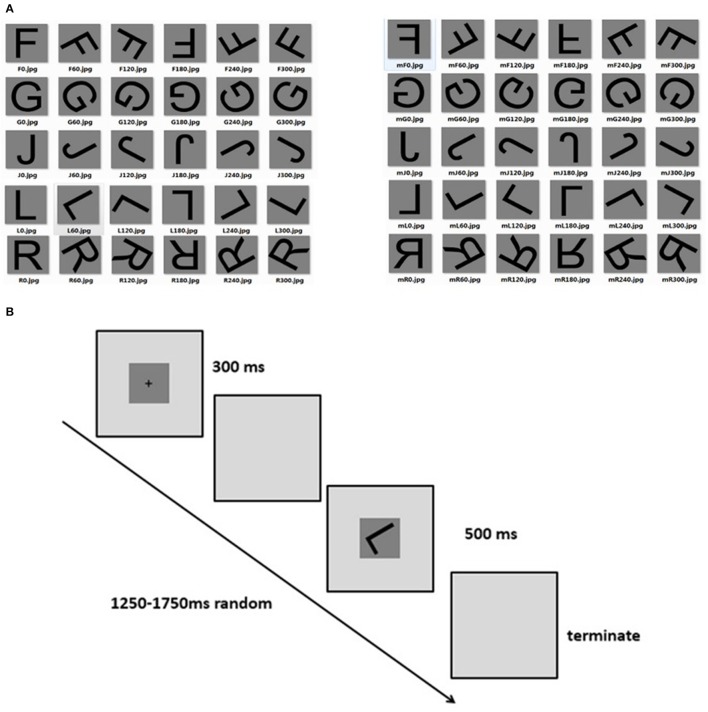
**(A)** Stimulus of mental rotation task (normal and mirror letters). **(B)** Procedure of mental rotation task.

Subject's task was to perform object mental rotation test on a computer. The stimulus of letter pictures is shown in Figure 1, with letters “F,” “G,” “J,” “L,” and “R” being chosen. There were six kind of orientation (0°, 60°, 120°, 180°, 240°, 300°) for normal and mirror letters. Tasks in the trial for the subjects were mental rotation of the stimulus into upright position and judge whether the stimulus is normal letter or not. Before the experiment, subjects were given written instructions and got acquainted with each task in a series of practice trials. At the beginning of each trial, a cross was presented in the center of the screen for a random duration (the duration ranging from 200 to 500 ms) using Eprime 2.0, a psychological experimental software (PST Co., Ltd; America). In rapid sequence the stimulus was presented for the subjects to make judgments and then click the mouse, with the stimulus presenting time last for 500 ms (interval of stimulus: 1,250 ~ 1,750 ms; Figure 1). Except for 0 and 180° stimuli which were repeated 60 times, other types of stimulus were all repeated for 30 times. The sequences of trials were randomly organized by the computer. There were 4 blocks in each task, with 100 trails, spacing between 2 and 3 min. The whole procedure of experiment was conducted in a darkened sound-attenuated EEG recording chamber.

#### EEG recording and analysis

EEG was recorded continuously using NeuroLab® Digital Amplifier by a set of 32 Ag/AgCl electrodes placed according to the 10–20 system. The EEG recording sites were FP1, FP2, F7, F3, Fz, F4, FT7, FC3, FCz, FC4, FT8, T7, C3, Cz, C4, T8, TP7, CP3, CPz, CP4, TP8, P7, P3, Pz, P4, P8, O1, Oz, and O2. EOG was recorded by electrodes placed on the bilateral external canthi and the left infraorbital and supraorbital areas. The tip of the nose was chosen as reference during recording. Electrode impedance was kept below 5 kΩ.

The rightly responded trials were accepted for further analysis. The reaction time (RT) between stimulus onset and response was averaged across clockwise and counter clockwise orientation angles. Previous studies have shown that there were no statistical differences between counter- and clockwise stimulus orientations in the same angle (Dalecki et al., 2012). The four conditions are: stimuli not rotated (0°), 60° (included stimuli rotated 60/300°), 120° rotation (included stimuli rotated 120/240°) and 180° rotation. Linear regression analysis on raw RTs as a function of the angle of rotation (angle of rotation ranging from 0 to 180°) was carried out. After the analysis we got the slope of regression line. While rotated the same orientations, the higher slope meant the subject needed more time to complete the mental rotation process. On the other hand, the inverse of the slope corresponds to the velocity of the mental rotation, that is, if a subject has a higher slope in the regression analysis, he ought to have a slower velocity of the mental rotation.

EOG artifacts were corrected using a correlation method proposed by Semlitsch et al. (1986). Following the artifact correction, the raw EEG was referenced to a whole head averaged reference spacing between ms and the preceding number. Then, the EEG was segmented in epochs of 1,000 ms, beginning 200 ms prior to stimulus onset and averaged. The amplitudes of P300 always appear over the parietal region after stimuli onset, and will overlap with RRN. So ERP amplitude and latency were detected from 300 to 500 ms, and Pz electrode was analyzed as an example. To explore the changing trend of the MR ability during simulated microgravity condition and different rotation angle, descriptive analysis and repeated measure ANOVA were performed.

## Results

**Figure 2:**
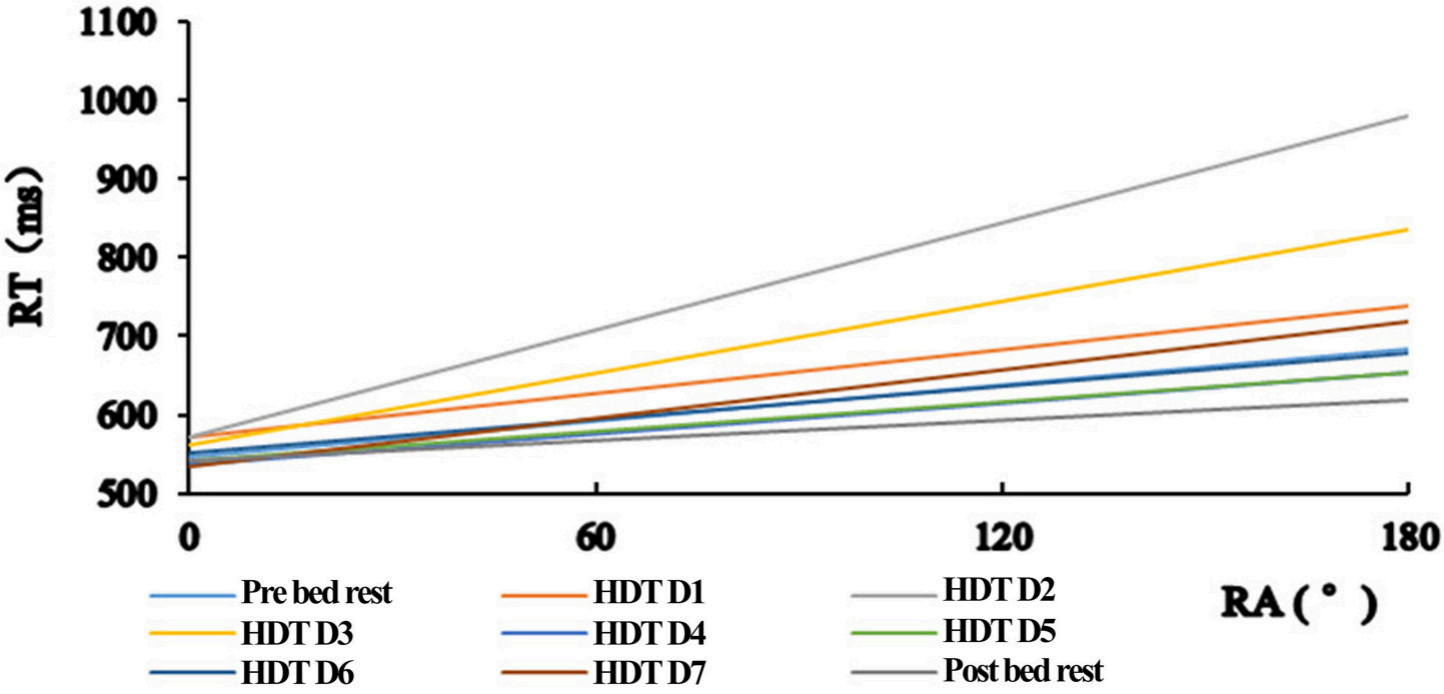
Liner regression of reaction time and rotation angle.

The accuracy rate of Pre bed rest and HDT D1 are 88 and 87%, and the rates of the rest 7 days are accordantly 86%. There is no significant difference in mean accuracy during simulated microgravity (*F* = 0.497, *p* = 0.875). Regression analysis suggests that there is significant linear trend between RT and rotation angle (RA) within all subjects (*p* < 0.05), thus show the slopes of the regression lines are valid. Under the condition of simulated microgravity, the overall RT of all subjects increased with the angle of stimulus increased. The slope of mental rotation changed from large to small are HDT D2, HDT D3, HDT D1, HDT D7, Pre bed rest, HDT D6, HDT D4, HDT D5, and Post bed rest. Intercept and slope showed no significant difference across the whole experiment period (Intercept: *F* = 1.028, *p* = 0.418; Slope: *F* = 13.000, *p* = 0.076). The biggest slope was observed in HDT D2 (1.08 ± 0.07 ms/°), and the smallest was in Post bed rest (0.95 ± 0.15 ms/°). The mean slope was first increased and then decreased, with smaller changing slope in Post bed rest (0.95 ± 0.15) than Pre bed rest (1.06 ± 0.37). The biggest intercept was shown in HDT D1 (552.08 ± 47.85 ms) and smallest in HDT D7 (530.13 ± 38.37 ms), with smaller scope in Post bed rest (543.89 ± 25.97 ms) than Pre bed rest (544.16 ± 35.93 ms, Figure 2).

**Figure 3:**
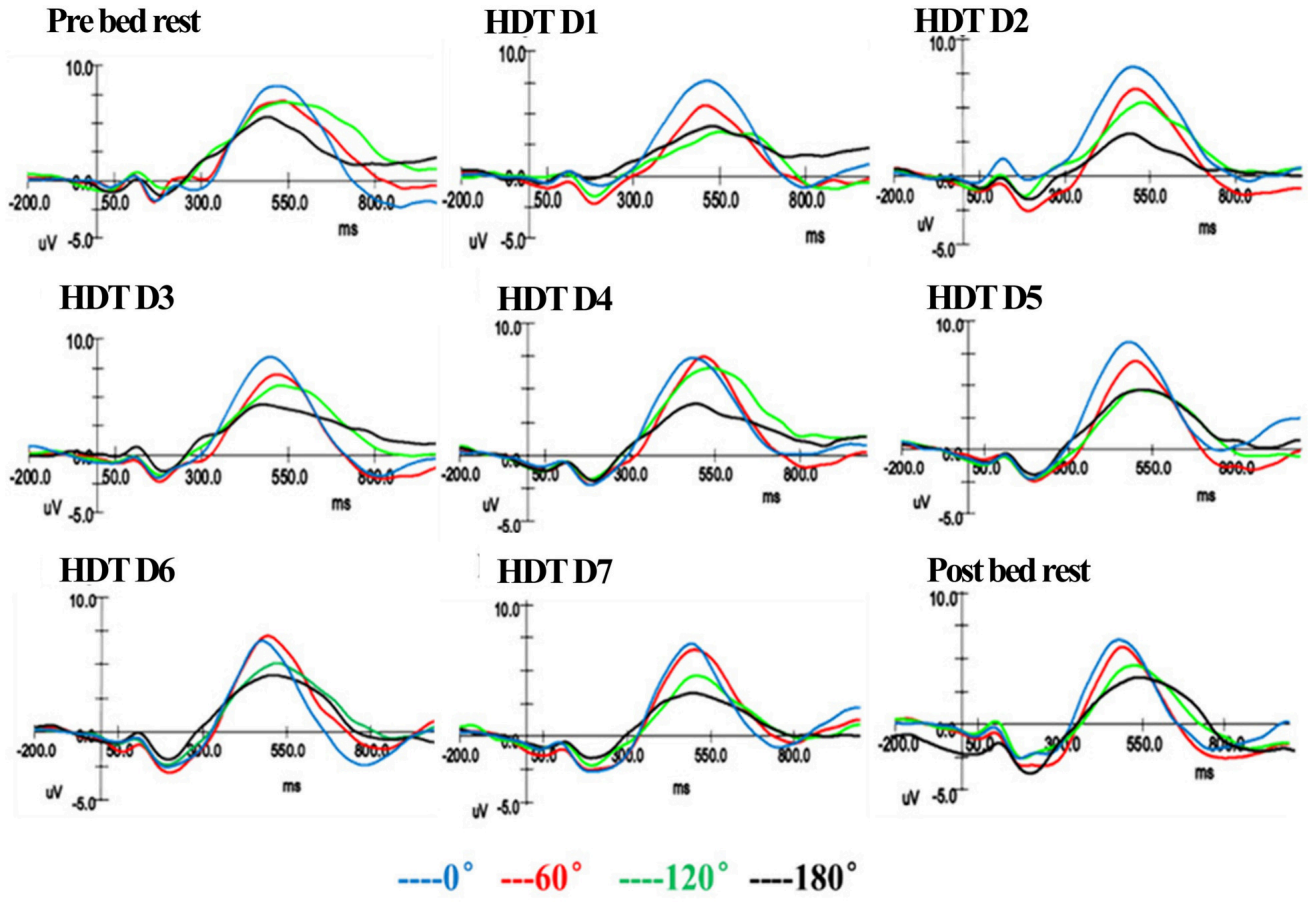
P300 of MR task across the experimental period.

Figure 3 showed the grand-average ERPs for each orientation of normal and mirror reversed letters at Pz, from which evident orientation effect was displayed. The rotation-related negativity becomes more negative with the increasing angular disparity from upright angle, with the effect being more evident for larger angle.

Repeat measure ANOVA of Amplitude showed that there is no interaction between RA and Time (*F* = 1.357, *p* > 0.05). The ANOVA showed significant main effects of different RA (*F* = 18.615, *p* < 0.001). Multiple comparisons (corrected by Bonferroni) were taken to compare the difference between of Amplitude of different RA. RA 0° (7.49 ± 0.51 μV, *p* = 0.002), RA 120° (6.43 ± 0.38 μV, *p* = 0.001) and RA 180° (5.89 ± 0.40 μV, *p* < 0.001) were all proved to have statistical difference with RA 0° (8.85 ± 0.52 μV).

**Figure 4:**
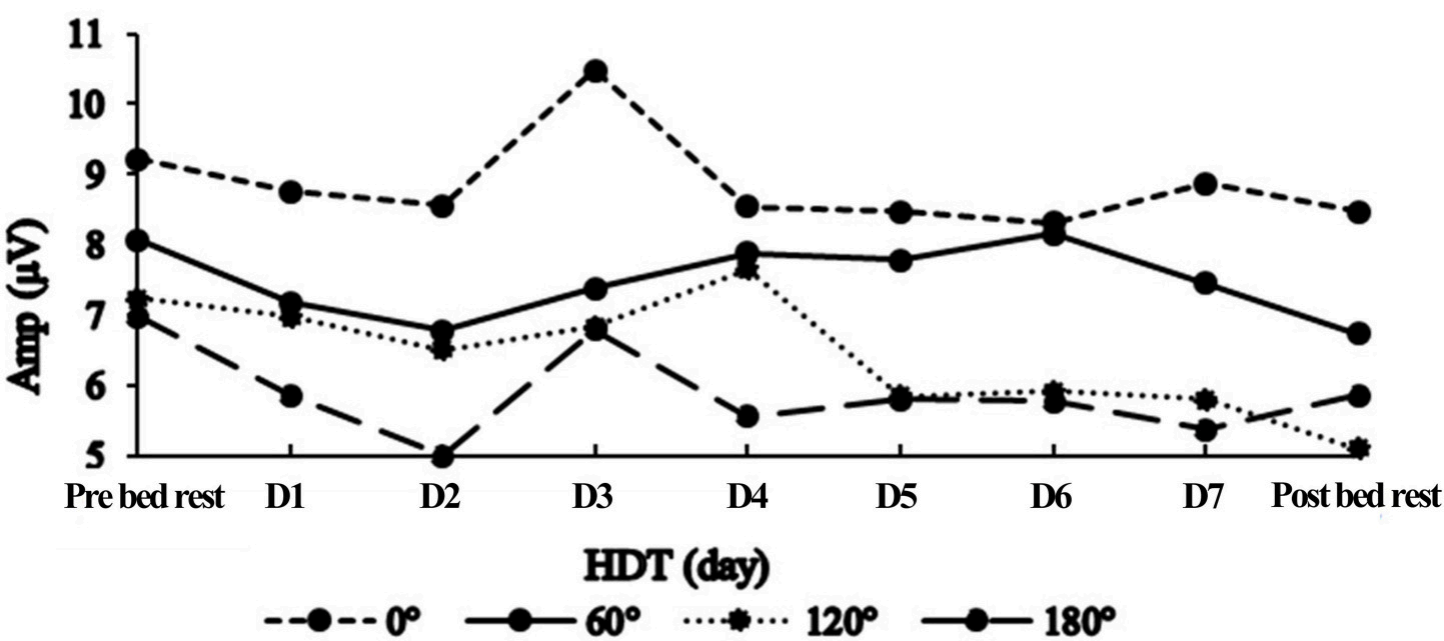
The amplitude of P300 in different rotation angle across the experiment period.

The ANOVA of Amplitude also showed significant main effects of Time (*F* = 2.658, *p* = 0.009). Following multiple comparisons (corrected by Bonferroni) shown that there is no significant difference between amplitude of different experiment days. Multiple comparisons (corrected by LSD) shown that HDT D2 (6.71 ± 0.41 μV, *p* = 0.008), HDT D5 (6.97 ± 0.42 μV, *p* = 0.037), HDT D7 (6.87 ± 0.43 μV, *p* = 0.015) and Post bed rest (6.54 ± 0.65 μV, *p* = 0.010) were proved to have statistical difference with Pre bed rest (7.87 ± 0.44 μV).Our previous research has revealed that male's mental rotation ability first decreased and then increased under the condition of 3-day simulated microgravity. To learn the time trend of ERP amplitude change, we separated the whole experimental process into two phases. The first phase is Pre bed rest to HDT D3. The second phase is HDT D4-D7. In the first phase, the amplitude decreased in the first 2 days during HDT and recovered slightly in HDT D3, the change trend in this phase is a “U” shape (Figure 4). Put Pre bed rest into together, the change trend of amplitude in the first phase show a “U” shape. In the second phase, there were no significant difference among amplitude of HDT D4-D7 (*p* > 0.05), the time trend of amplitude change in this phase is fluctuation in a same level.

## Discussion

In the present study, 20 healthy males participated in a 7-day MR task under −6° HDT model. Behavioral data shows that the accuracy at baseline was higher than HDT period and post HDT period. This finding indicated that simulated microgravity may have negative effects on people's MR ability. Linear regression analysis showed that RT is in significantly liner relationship with RA. As RT is prolonged along with the increasing RA, which is consistent with previous research (Bryden et al., 1990), we may draw the conclusion that simulated microgravity hasn't changed the basic regularity of mental rotation.

The slope of the regression equation was related to the mental rotation speed according to the results of regression analysis with RA as an independent variable and reaction time as dependent variable (Shepard and Judd, 1976). Despite that the difficulty of letter rotation differs in this study, research shows that the rotation speed of slope is not affected by the difficulty or ease of experimental materials (Wiedenbauer and Jansen-Osmann, 2008). In the period of pre-, during and post-HDT, the individual's mental rotation processing speed from high to low were HDT, HDT, D1, D2, HDT D3 HDT D7, Test the Pre, HDT D4, HDT D5, and Post bed rest. The rotation speed first increased and then decreased, and was slower than baseline after HDT.

**Figure 5:**
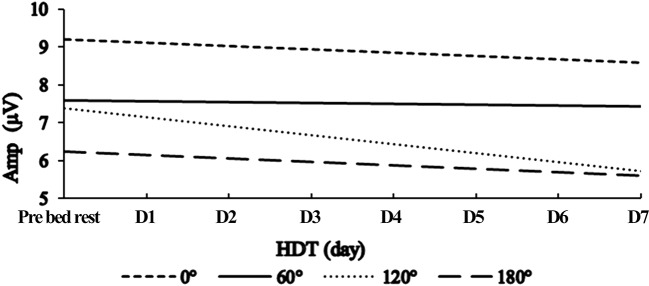
Liner regression of Amplitude of P300 and Time.

The rotation-amplitude-related negativity shows the depth of the mental rotation process, and it becomes larger when RA increases (Heil et al., 1998). This scientific judgement has been proved by the results of this study. After simulated microgravity, the amplitude was smaller than the baseline. In HDT D2 the amplitude of four angles was the smallest, and then back to the baseline in HDT D3, which is exactly the same result with our previous study (Liao et al., 2013; Zhu et al., 2013). At the early stage of simulated microgravity, individual's mental rotation ability first decreased then increased, changing like the shape of an “U” shape. This result indicated that inhibition or damage may occur to individual brain function in the early stage of simulated weightlessness, and recovery may follow as the simulated weightlessness lasts. This result indicated that the brain blood flow changes during HDT may suppress individual's MR ability. As HDT time lasts, individual gradually adapt to the changes of the brain, thus after suppression, brain activity began to recover. However, according to our research results, the MR ability still went down compared to the baseline. To analyze the changing pattern of amplitude, linear regression was conducted (Figure 5). It was demonstrated that the amplitude of RA180 and RA120° had a linear downward trend caused by simulated weightlessness, while no linear relationship of amplitude of RA0 and RA60° with HDT time was found, indicating that simulated weightlessness has different effects on individuals' spatial representation transformation ability when they rotate different angles of stimulus in mind. There is no linear change in the ability of rotating acute angle stimulus, while the individual MR ability of obtuse and upside-down stimuli is on a linear decrease during the HDT period.

There are two possible explanations for the time series of MR ability changes observed in the current study. First, the MR ability change is mainly due to psychological effects caused by stressors. Under the condition of simulated weightlessness, subjects faced multiple stressors along with environment change. Thus, the individual's MR ability changes go through three phases as the stress response curve, namely, the alert stage when MR ability went down; the resistance stage when MR ability recovered and rose, and the fatigue stage when MR ability went down again. Three days later, the individual's MR ability randomly fluctuated. The changing degree varied according to different individual adaptability. The second explanation is that the influence of simulated weightlessness on mental rotation ability is periodic, and it is mostly due to physiological effects induced by the body fluid redistribution. The first period is 3 days. MR ability in this period will show changes in regular patter of decrease-increase-decrease. The second period is 3 days or perhaps longer and MR ability fluctuates randomly. Along with the result from our previous fMRI study, a self-adaption change in brain function may account for the MR ability change in the current study, and it perhaps result for a mixed effects in psychological and physiological levels.

To sum up, 7-day simulated weightlessness does not change the basic process of mental rotation. The overall changing rate of processing speed has shown a down-and-up trend. After HDT, the mental rotation speed is slower than the baseline. The influence of simulated weightlessness on individual's mental rotation ability reveals a neural adaptive mechanism. In the first 3 days of simulated weightlessness, the MR ability first decreased and then went back, changing like a “U” shape. In the rest days it went into a state of random fluctuations. The time series of MR ability changes observed in the current HDT study could help us clarify the performance damage occurred in the early stage of spaceflight. As indicated by the current result, the first 3 days may be the critical period for performance maintain during spaceflight.

## Author contributions

Authors XL and HL conceived and designed the experiments. Authors HW, JD, YL, and CW performed the experiments. JD analyzed the data. JD contributed materials and analysis tools. Authors HW and YL wrote the manuscript and contributed equally to this work.

### Conflict of interest statement

The authors declare that the research was conducted in the absence of any commercial or financial relationships that could be construed as a potential conflict of interest.

## References

[B1] Annett M. (1970). A classification of hand preference by association analysis. Br. J. Psychol. 61, 303–321. 10.1111/j.2044-8295.1970.tb01248.x 5457503

[B2] Benke T. Koserenko O. Watson N. Gerstenbrand F. (1993). Space and cognition: the measurement of behavioral functions during a 6-day space mission. Aviat. Space Environ. Med. 64, 376–379. 8503810

[B3] Bryden M. George J. Inch R. (1990). Sex differences and the role of figural complexity in determining the rate of mental rotation. Percept. Mot. Skills 70, 467–477. 10.2466/pms.1990.70.2.467 2342846

[B4] Cooper L. A. Shepard R. N. (1973). Chronometric Studies of the Rotation of Mental Images. New York, NY: Academic Press.

[B5] Cooper L. A. Shepard R. N. (1975). Mental transformation in the identification of left and right hands. J. Exp. Psychol. Hum. Percept. Perform. 1, 48–56. 10.1037/0096-1523.1.1.48 1141835

[B6] Dalecki M. Hoffmann U. Bock O. (2012). Mental rotation of letters, body parts and complex scenes: separate or common mechanisms? Hum. Mov. Sci. 31, 1151–1160. 10.1016/j.humov.2011.12.001 22244585

[B7] Eddy D. R. Schiflett S. G. Schlegel R. E. Shehab R. L. (1998). Cognitive performance aboard the life and microgravity spacelab. Acta Astronaut. 43, 193–210. 10.1016/S0094-5765(98)00154-4 11541924

[B8] Grigoriev A. I. Egorov A. D. (1992). General mechanisms of the effect of weightlessness on the human body. Adv. Space Biol. Med. 2, 1–42. 10.1016/S1569-2574(08)60016-7 1364147

[B9] Heil M. (2002). The functional significance of ERP effects during mental rotation. Psychophysiology 39, 535–545. 10.1111/1469-8986.3950535 12236320

[B10] Heil M. Rauch M. Hennighausen E. (1998). Response preparation begins before mental rotation is finished: evidence from event-related brain potentials. Acta Psychol. 99, 217–232. 10.1016/S0001-6918(98)00012-2 9708033

[B11] Hirayanagi K. Kamiya A. Iwase S. Mano T. Sasaki T. Oinuma M.. (2004). Autonomic cardiovascular changes during and after 14 days of head-down bed rest. Auton. Neurosci. 110, 121–128. 10.1016/j.autneu.2004.01.001 15046736

[B12] Horst A. C. Jongsma M. L. Janssen L. K. Lier R. Steenbergen B. (2012). Different mental rotation strategies reflected in the rotation related negativity. Psychophysiology 49, 566–573. 10.1111/j.1469-8986.2011.01322.x 22091978

[B13] Hu W. Lu Y. Ren C. Zhang J. X. (2013). ERP evidence for the time course of mental rotation in the mirror reading of Chinese words. Neurosci. Lett. 552, 151–155. 10.1016/j.neulet.2013.07.041 23933203

[B14] Huang C. F. Liu C. J. (2012). An event-related potentials study of mental rotation in identifying chemical structural formulas. Eur. J. Educ. Res. 1, 37–54.

[B15] Johnston R. S. Dietlein L. F. (1977). Biomedical Results From Skylab. NASA SP-377, Biomedical Results from Skylab. 11977284

[B16] Jolicoeur P. (1985). The time to name disoriented natural objects. Mem. Cogn. 13, 289–303. 10.3758/BF03202498 4079746

[B17] Jolicoeur P. (1988). Mental rotation and the identification of disoriented objects. Can. J. Psychol. Rev. 42, 461–478. 3268325 10.1037/h0084200

[B18] Jolicoeur P. (1990). Identification of disoriented objects: a dual-systems theory. Mind Lang. 5, 387–410. 10.1111/j.1468-0017.1990.tb00170.x

[B19] Kelly T. H. Hienz R. D. Zarcone T. J. Wurster R. M. Brady J. V. (2005). Crewmember performance before, during, and after spaceflight. J. Exp. Anal. Behav. 84, 227–241. 10.1901/jeab.2005.77-04 16262187 PMC1243980

[B20] Koga K. (2000). Gravity cue has implicit effects on human behavior. Aviat. Space Environ. Med. 71, 78–86. 10993315

[B21] Kornilova L. (1997). Orientation illusions in spaceflight. J. Vestib. Res. 7, 429–440. 10.1016/S0957-4271(96)00184-X 9397393

[B22] Leone G. (1998). The effect of gravity on human recognition of disoriented objects. Brain Res. Rev. 28, 203–214. 10.1016/S0165-0173(98)00040-X 9795218

[B23] Liao Y. Miao D. Huan Y. Yin H. Xi Y. Liu X. (2013). Altered regional homogeneity with short-term simulated microgravity and its relationship with changed performance in mental transformation. PLoS ONE 8:E64931. 10.1371/journal.pone.0064931 23755162 PMC3670926

[B24] Manzey D. Lorenz B. (1998). Mental performance during short-term and long-term spaceflight. Brain Res. Rev. 28, 215–221. 10.1016/S0165-0173(98)00041-1 9795225

[B25] McIntyre J. Berthoz A. Lacquaniti F. (1998). Reference frames and internal models for visuo-manual coordination: what can we learn from microgravity experiments? Brain Res. Rev. 28, 143–154. 10.1016/S0165-0173(98)00034-4 9795191

[B26] Morphew M. E. (2001). Psychological and human factors in long duration spaceflight. McGill J. Med. 6, 74–80.

[B27] Newberg A. Alavi A. (1998). Changes in the central nervous system during long-duration space flight: implications for neuro-imaging. Adv. Space Res. 22, 185–196. 10.1016/S0273-1177(98)80010-0 11541396

[B28] Palinkas L. A. (2007). Psychosocial issues in long-term space flight: overview. Gravit. Space Res. Biol. Bull. 14, 25–33. 11865866

[B29] Peronnet F. Farah M. J. (1989). Mental rotation: an event-related potential study with a validated mental rotation task. Brain Cogn. 9, 279–288. 10.1016/0278-2626(89)90037-7 2923718

[B30] Pozzo T. Papaxanthis C. Stapley P. Berthoz A. (1998). The sensorimotor and cognitive integration of gravity. Brain Res. Rev. 28, 92–101. 10.1016/S0165-0173(98)00030-7 9795160

[B31] Riečanský I. Jagla F. (2008). Linking performance with brain potentials: mental rotation-related negativity revisited. Neuropsychologia 46, 3069–3073. 10.1016/j.neuropsychologia.2008.06.016 18639565

[B32] Riečanský I. Tomova L. Katina S. Bauer H. Fischmeister F. P. Lamm C. (2013). Visual image retention does not contribute to modulation of event-related potentials by mental rotation. Brain Cogn. 83, 163–170. 10.1016/j.bandc.2013.07.011 23994461

[B33] Sandal G. M. (2007). Psychosocial issues in space: future challenges. Gravit. Space Biol. Bull. 14, 47–54. 11865868

[B34] Schendan H. E. Lucia L. C. (2009). Visual object cognition precedes but also temporally overlaps mental rotation. Brain Res. 1294, 91–105. 10.1016/j.brainres.2009.07.036 19631629

[B35] Semlitsch H. V. Anderer P. Schuster P. Presslich O. (1986). A solution for reliable and valid reduction of ocular artifacts, applied to the P300 ERP. Psychophysiology 23, 695–703. 10.1111/j.1469-8986.1986.tb00696.x 3823345

[B36] Shehab R. L. Schlegel R. E. (2000). Applying quality control charts to the analysis of single-subject data sequences. Hum. Fact. J. Hum. Fact. Ergon. Soc. 42, 604–616. 10.1518/001872000779698033 11324853

[B37] Shepard R. N. Judd S. A. (1976). Perceptual illusion of rotation of three-dimensional objects. Science 191, 952–954. 10.1126/science.1251207 1251207

[B38] Shepard R. N. Metzler J. (1971). Mental rotation of three-dimensional objects. Science 171, 701–703. 10.1126/science.171.3972.701 5540314

[B39] Tarr M. J. Pinker S. (1989). Mental rotation and orientation-dependence in shape recognition. Cogn. Psychol. 21, 233–282. 10.1016/0010-0285(89)90009-1 2706928

[B40] Wiedenbauer G. Jansen-Osmann P. (2008). Manual training of mental rotation in children. Learn. Instruct. 18, 30–41. 10.1016/j.learninstruc.2006.09.009

[B41] Wijers A. A. Otten L. J. Feenstra S. Mulder G. Mulder L. J. (1989). Brain potentials during selective attention, memory search, and mental rotation. Psychophysiology 26, 452–467. 10.1111/j.1469-8986.1989.tb01951.x 2798695

[B42] Wraga M. Boyle H. K. Flynn C. M. (2010). Role of motor processes in extrinsically encoding mental transformations. Brain Cogn. 74, 193–202. 10.1016/j.bandc.2010.07.005 20888110

[B43] Zacks J. M. Vettel J. M. Michelon P. (2003). Imagined viewer and object rotations dissociated with event-related fMRI. J. Cogn. Neurosci. 15, 1002–1018. 10.1162/089892903770007399 14614811

[B44] Zayzafoon M. Meyers V. E. McDonald J. M. (2005). Microgravity: the immune response and bone. Immunol. Rev. 208, 267–280. 10.1111/j.0105-2896.2005.00330.x 16313354

[B45] Zhu T. Zhang Q. Duan J. Liao Y. Liu X. (2013). Effect of short-term simulated microgravity on man's ability in object mental rotation test. Acta Acad. Med. Militaris Tertiae 35, 2151–2154.

